# The Potential of Phage Therapy in Sepsis

**DOI:** 10.3389/fimmu.2017.01783

**Published:** 2017-12-11

**Authors:** Andrzej Górski, Ewa Jończyk-Matysiak, Marzanna Łusiak-Szelachowska, Ryszard Międzybrodzki, Beata Weber-Dąbrowska, Jan Borysowski

**Affiliations:** ^1^Laboratory of Bacteriophages, Hirszfeld Institute of Immunology and Experimental Therapy, Polish Academy of Sciences (HIIET PAS), Wrocław, Poland; ^2^Department of Clinical Immunology, Transplantation Institute, Medical University of Warsaw, Warsaw, Poland

**Keywords:** phages, sepsis, immunomodulation, immunodeficiency, application of phages

## Abstract

Sepsis remains a difficult clinical challenge, since our understanding of its immunopathology is incomplete and no efficacious treatment currently exists. Its earlier stage results from an uncontrolled inflammatory response to bacteria while in the later stage disturbed immune response with immunodeficiency syndrome develops. More than a hundred of clinical trials have not provided an efficient therapy which could ascertain an improvement or cure. Recent advancements in immunobiology of bacterial viruses (phages) indicate that in addition to their well-known antibacterial action phages have potent immunomodulating properties. Those data along with preliminary observations in experimental animals and the clinic strongly suggest that clinical trials on the efficacy of phages in sepsis are urgently needed.

Sepsis is the leading cause of death in critically ill patients. More than 30 million cases of sepsis occur worldwide each year, and it is increasing 9–13% annually with a mortality rate of approximately 33% (1). Sepsis is considered to be an uncontrolled inflammatory response to bacterial infection associated with immunosuppression, an inability to clear infection and predisposition to nosocomial infections. Excessive release of oxidants and proteases by neutrophils is responsible for associated organ injury, especially in the lungs, as intrapulmonary sequestration of neutrophils and acute respiratory distress syndrome are its frequent complications (2), while the abdomen and urinary tract are also often affected (1). Also, exacerbated release of inflammatory cytokines and the resulting hyperactivation of immune cells (“cytokine storm”) appears to be important contributing factors. Although no single mediator or pathogen is responsible for the pathophysiology of sepsis, bacterial toxins (especially endotoxins) play a pivotal role in this disorder. In the past 25 years, the definitions of sepsis and septic shock and concepts of their pathophysiology have been hotly debated, with no gold standard achieved (3). Given the complexity of sepsis pathophysiology and its non-specificity with regard to infection, more than a hundred clinical trials have been carried out targeting the host response to infection, yet none of them has yielded a reliable treatment modality assuring clear clinical efficacy (1). Therefore, the development of new treatment modalities to prevent the progression of initial stages of sepsis to multiorgan failure and death is urgently needed (4). Host-directed therapy (HDT) is an emerging concept in anti-infective therapy meant to counteract the action of host factors required by pathogens for replication and persistence, to stimulate protective immunity, and to achieve a balance of immune reactivity to pathogens. Its major strategy is to upregulate the activity of phagocytes and limit inflammation (5). This strategy is supported by observations indicating that neutrophils may play a beneficial and deleterious role in the outcome of sepsis (6).

In the past decade, our understanding of bacteriophages (phages) and their current and potential position in microbiology and immunobiology has undergone major changes and has gained a new dimension. The original concept of phage highlighted its well-known antibacterial action, and the resulting practical therapeutic implications included the use of phage in antimicrobial therapy—especially in antibiotic-resistant infections. Given the increasing crisis of antimicrobial resistance, the interest in phage therapy has increased tremendously as more data suggesting its safety and efficacy have accumulated. Nevertheless, data showing the ability of phages to interact with the immune system and to modify its functions strongly suggest that phages may cause immunomodulating effects which can have practical implications for therapy. Of particular interest are the following immunomodulating activities of phages:

(1) Phages show strong anti-inflammatory properties that may be independent of their well-known antibacterial activities. The possible mechanisms responsible for this effect may involve LPS binding, inhibition of excessive reactive oxygen species production and induction of IL-10 production. It has been shown that phages and their proteins may diminish inflammatory infiltration induced by endotoxin and allogeneic transplantation, and lower clinical indices of inflammation in patients on phage therapy (CRP, sedimentation rate, leukocytosis). In fact, a decrease of CRP in some patients may be very significant even though eradication of infections has not been achieved (7, 8).
(2) Phages do not activate murine and human dendritic cells and may inhibit skin allograft rejection in normal and presensitized mice as well as dampen autoimmune reaction in a mouse model of collagen-induced arthritis (9, 10). They induce the anti-inflammatory IL-1 receptor antagonist (IL-1RA) synthesis by human mononuclear cells, a cytokine blocking the expression of pro-inflammatory cytokines and inhibiting the activation of Th1 cells and macrophages (11). Interestingly, IL-1RA blockade was associated with significant improvement in survival of patients with sepsis (12). Recent data confirm that the administration of recombinant IL-1RA can be beneficial in septic patients in whom its adequate levels have been achieved (13). Phages may also downregulate the expression of TLR4 (whose activation induces secretion of pro-inflammatory cytokines) (11). Interestingly, it was recently demonstrated that in a model of post-septic mice TLR4 deficiency improves immune paralysis; therefore, modulation of TLR4 activity may be useful in treating sepsis (14). Phages may also interact with platelets (PLT), which may be viewed as “the underappreciated orchestrator of the immune system” (15). PLT may participate in the leukocyte recruitment leading to increased severity of inflammation and their aggregation in this condition in response to agonist is amplified (15). Phages inhibit platelet (PLT) adhesion to fibrinogen and cause some reduction of T cell adhesion to that protein. In addition, thrombin-induced PLT aggregation *in vitro* may be decreased (9). PLT role in host response to sepsis has been subject of intense research (16) and more studies are needed to determine if indeed phage interactions with PLT may contribute to beneficial effects in sepsis.
  Phage therapy may lead to downregulation of excessive immune responses, thus contributing to maintenance of immune homeostasis. This mode of action may depend on the initial immune status of an individual causing upregulation when the immune response is depressed and downregulation when it is hyperactive: for example, abnormal B cell function returned to normal values in patients on phage therapy (7). Furthermore, phage therapy-dependent upregulation of cytokine production when it was initially low and lowering in patients in whom it was initially high was also noted (17). Recent reviews have described immunomodulating activities of phages in detail (7, 9, 18).
(3) In almost 50% of patients on phage therapy, an increase in phagocytosis was noted which was associated with good clinical outcome. This observation seems to be a strong argument for phage treatment of sepsis: as noted, stimulation of phagocytosis is recommended as an important part of anti-sepsis strategy (5). Phages do not induce granulocyte degranulation, an effect which could contribute to further tissue injury (19). When confronted with bacteria phages facilitate microbial phagocytosis by human granulocytes (20). In this regard, it should be noted that the discoverer of phages, d’Herelle, determined that phages act as specific opsonins markedly facilitating bacterial phagocytosis (21).

Our data strongly suggest that phage therapy does not impair human granulocyte and monocyte ability to kill invading and standard strain bacteria and may even correct monocyte deficiency in patients with urinary tract infections treated with phages (22). The observed amelioration of phagocytosis reflects the outcome of a variety of factors influencing phagocyte functions in patients on phage therapy, one of the most important being pathogen burden. It is well known that pathogens have developed countermeasures to avoid detection, thereby impairing signaling and paralyzing machinery underlaying phagocytosis and that neutrophil functions are depressed in patients with chronic infections. This deficiency may result from pathogen activity and therapy (e.g., antibiotics) (23, 24). Other authors have shown that neutrophils are required to control both phage-sensitive and phage-resistant pathogens; importantly, phages alone were unable to clear bacterial infection in neutrophil-depleted mice. Thus, the success of phage therapy depends on synergistic activities of phagocytes and phages, a phenomenon referred to as “immunophage synergy” (25). In accord with this assumption, reducing pathogen burden by phages may contribute to alleviating pathogen-dependent impairment of phagocyte functions. Evidently, those activities of phages in combination with their antibacterial functions strongly suggest that they could be of value in the treatment of sepsis. In particular, early phage administration could help eradicate infection, limit inflammation, and upregulate phagocytosis (which are the key recommendations of the HDT and may prevent the development of full-blown sepsis with resulting organ failure and death) (5). Recent data indicate that phages may induce IL-10 production by human mononuclear cells (11). IL-10 has been recognized as a potent anti-inflammatory cytokine limiting cell and tissue injury during bacterial infections. In a rat model of sepsis-induced acute kidney injury, upregulating IL-10 expression by macrophages was associated with attenuation of sepsis (26). In a positive feedback loop, IL-10 may also induce this cytokine in neutrophils, both *in vitro* and *in vivo* (27). Using a whole blood assay simulating the *in vivo* situation it was shown that IL-10 downregulates neutrophil phagocytosis of bacteria (28) confirming earlier data (29). On the other hand, IL-10 may upregulate phagocytosis in monocytes (28, 30). Therefore, IL-10 may have opposing effect on different phagocyte populations. Excessive secretion of IL-10 may contribute to immunosuppression typical for later stages of sepsis (31). Therefore, phage treatment of later stages of the syndrome may be more problematic when ensuing immunosuppression prevails. However—as pointed out previously—phages have been demonstrated to upregulate *in vivo* and *in vitro* cytokine production in patients with immunodeficiency; moreover, available data suggest that phage therapy is safe also in animals and patients with various forms of immunodeficiency syndrome (19). Table 1 shows the possible influence of phages on immune response during early and advanced stage of sepsis.

**Table 1:** Phage-induced immunomodulation at the early and advanced stage of sepsis.

Sepsis	
Early stage	Advanced stage
↑ Production of IL-10	↑ Immunomodulation
↓ ROS production	↓ ROS production
↓ Inflammatory infiltration	↓ Inflammatory infiltration
↑ Phagocytosis	↑ Phagocytosis
↑ Intracellular killing	↑ Intracellular killing
↑ LPS-binding	Lack of granulocyte degranulation

*↑, increase; ↓, decrease*.

*Phage therapy may induce various types of immunomodulation in consecutive stages of sepsis*.

Interestingly, the potential application of phages (and their enzymes, lysins) in the treatment of sepsis has already received some experimental support, both in experimental animals and the clinic. Table 2 presents the reported data on phage/lysin efficacy in the treatment of sepsis caused by different pathogens (32–49). In addition, there are initial clinical observations supporting the experimental data. We have reported good results in sepsis patients when phages were administered orally (50). Phages can translocate from the gastrointestinal tract; in addition, this route of administration was also found to be efficient in the mouse model (34).

**Table 2:** Bacteriophages and phage lysins in the treatment of sepsis.

Antibacterial agent bacteriophage (B) or lysin (L)	Animal model	Pathogen	Route/time of phage/lysin administration (bold font—the best protection)	Reference
B	Mouse model	*Acinetobacter baumanii*	i.p./2 h postinfection, **up to 7 days postinfection**	(32)
B	Mouse model	*Staphylococcus aureus*	**i.p./6 h postinfection**	(33)
B	Mouse model	*Klebsiella pneumonia* NK-5	**i.p./30 min postinfection**	(34)
			**Intragastric/30 min postinfection**	
B	Mouse model	*S. aureus*	i.p./**30 min postinfection**; in delayed treatment 0, 1, 2, 3, **4**, 6 h postinfection	(35)
B	Mouse model	*Pseudomonas aeruginosa* MDR	**i.p./45 min postinfection**	(36)
B	Mouse model	*P. aeruginosa*	*per os*/1 day before, **1 day**, 6 days postinfection	(37)
			i.p./1 day before or **simultaneously** with strain	
B	Mouse model	*P. aeruginosa* IMPR-Pa	i.p./**up to 1 h**; 3, 6 h postinfection	(38)
B	Mouse model	*Escherichia coli* ESBL (+)	i.p./**40 min, up to 60 min postinfection**	(39)
B	Mouse model	*K. pneumonia* MDR	**i.p./45 min postinfection**	(40)
B	Rat model	*E. coli* ESBL (+)	s.c./**7 h** and 24 h postinfection	(41)
B	Chickens, calves	*E. coli* K1	**i.m./simultaneously with strain (chickens)**	(42)
			**i.m./8 h postinfection (calves)**	
L	Mouse model	*Streptococcus pneumoniae*	**i.p./1 h postinfection**	(43)
L	Mouse model	*S. aureus* MR	**i.p./30 min postinfection**	(44)
L	Mouse model	*Streptococcus pyogenes*	**i.p./3 h postinfection**	(45)
		*S. aureus* MR		
L	Mouse model	*S. aureus* MR	**i.p./2 h postinfection**	(46)
L	Mouse model	*S. pneumoniae*	**i.p./1 h postinfection** (daptomycin + lysin)	(47)
L	Mouse model	*S. aureus* MR	**i.p./1 h postinfection**	(48)
L	Mouse model	*S. pneumoniae*	**i.p./1 h postinfection**	(49)

*i.p., intraperitoneal injection; s.c., subcutaneous injection; i.m., intramuscular injection*.

*Summary of the data showing the efficacy of phages and their lysins in the treatment of experimentally induced sepsis*.

In 2017, two cases of successful phage treatment of sepsis were reported. A patient with peritonitis, severe abdominal sepsis and renal insufficiency received phage against his *Pseudomonas aeruginosa* isolate intravenously. Immediately, blood cultures turned negative, CRP dropped and fever disappeared, and renal function recovered. Recently, a dramatic result was reported in a patient with sepsis in the course of necrotizing pancreatitis caused by *Acinetobacter baumannii*. Administration of phages intravenously and percutaneously into abscess cavities caused prompt clearance of infection, reversal of the patient’s downward clinical course and return to health (51). Those clinical data may suggest that phage therapy may also be efficient—at least in some cases—when administered at later stages of sepsis.

Recently, an important clinical trial evaluating the efficacy of phage therapy in urinary tract infection was announced which may indicate that further progress in the introduction of this treatment is on the horizon (52). The fact that practically all clinical trials reported so far failed to result in a really significant progress in the treatment of sepsis and the data reported in this article suggest that clinical trials on the efficacy of phage therapy in sepsis are urgently needed.

## Author Contributions

AG drafted the main part of the manuscript. EJ-M, MŁ-S, RM, BW-D, and JB contributed parts of the manuscript. All authors revised the manuscript.

## Conflict of Interest Statement

AG, RM, BW-D, and JB are co-inventors of patents owned by the Institute and covering phage preparations. Other authors declare that they have no conflict of interest.

## References

[1] Minasyan H Sepsis and septic shock: pathogenesis and treatment. J Crit Care (2017) 40:229–42. 10.1016/j.jcrc.2017.04.015 28448952

[2] Hotchkiss RS Karl IE The pathophysiology and treatment of sepsis. N Engl J Med (2003) 348:138–50. 10.1056/NEJMra021333 12519925

[3] Kalantari A Mallemat H Wiengart SD Sepsis definition: the search for gold and what CMS got wrong. West J Emerg Med (2017) 18:951–6. 10.5811/westjem.2017.4.32795 28874949 PMC5576633

[4] Hattorri Y Hattori K Suzuki T Matsuda N. Recent advances in the pathophysiology and molecular basis of sepsis-associated organ dysfunction: novel therapeutic implications and challenges. Pharmacol Ther (2017) 177:56–66. 10.1016/j.pharmthera.2017.02.040 28232275

[5] Kaufmann SHE Dorhoi A Hotchkiss RS Bartenschlager R. Host-directed therapies for bacterial and viral infections. Nat Rev Drug Discov (2017). 10.1038/nrd.2017.162 28935918 PMC7097079

[6] Sonego F Castanheira FV Ferreira RG Kanashiro A Leite CA Nascimento DC Paradoxical roles of the neutrophil in sepsis: protective and deleterious. Front Immunol (2016) 7:155. 10.3389/fimmu.2016.00155 27199981 PMC4844928

[7] Górski A Międzybrodzki R Borysowski J Dabrowska K Wierzbicki P Ohams M Phage as a modulator of immune responses: practical implications for phage therapy. Adv Virus Res (2012) 83:41–71. 10.1016/B978-0-12-394438-2.00002-5 22748808

[8] Międzybrodzki R Borysowski J Weber-Dabrowska B Fortuna W Letkiewicz S Szufnarowski K Clinical aspects of phage therapy. Adv Virus Res (2012) 83:73–121. 10.1016/B978-0-12-394438-2.00003-7 22748809

[9] Górski A Dabrowska K Międzybrodzki R Weber-Dabrowska B Łusiak-Szelachowska M Jończyk-Matysiak E Phages and immunomodulation. Future Microbiol (2017) 12:905–14. 10.2217/fmb-2017-0049 28434234

[10] Międzybrodzki R Borysowski J Kłak M Jończyk-Matysiak E Obmińska-Mrukowicz B Siszko A *In vivo* studies on influence of bacteriophage preparations on the autoimmune inflammatory process. Biomed Res Int (2017) 2017:9 10.1155/2017/3612015 PMC5671675 29201902

[11] Van Belleghem JD Clement F Merabishvilli M Lavigne R Vaneechoutte M. Pro- and anti-inflammatory responses of peripheral blood mononuclear cells induced by *Staphylococcus aureus* and *Pseudomonas aeruginosa* phages. Sci Rep (2017) 7:8004. 10.1038/s41598-017-08336-9 28808331 PMC5556114

[12] Shakoory B Carcillo JA Chatham WW Amdur RL Zhao H Dinarello CA Interleukin-1 receptor blockade is associated with reduced mortality in sepsis patients with features of macrophage activation syndrome: reanalysis of a prior phase III trial. Crit Care Med (2016) 44:275–81. 10.1097/CCM.0000000000001402 26584195 PMC5378312

[13] Meyer NJ Reilly JP Anderson BJ Palakshappa JA Jones TK Dunn TG Mortality benefit of recombinant human interleukin-1 receptor antagonist for sepsis varies by initial interleukin-1 receptor antagonist plasma concentration. Crit Care Med (2017). 10.1097/CCM.0000000000002749 PMC5734955 28991823

[14] Cao C Chai Y Shou S Wang J Huang Y Ma T Toll-like receptor 4 deficiency increases resistance in sepsis–induced immune dysfunction. Int Immunopharmacol (2017) 54:169–76. 10.1016/j.intimp.2017.11.006 29149705

[15] Ali RA Wuescher LM Worth RG. Platelets: essential components of the immune system. Curr Trends Immunol (2015) 16:65–78. 27818580 PMC5096834

[16] Graham SM Liles WC Platelets in sepsis: beyond hemostasis. Blood (2016) 127:2947–9. 10.1182/blood-2016-03-706168 27313324

[17] Weber-Dabrowska B Zimecki M Mulczyk M Effective phage therapy is associated with normalization of cytokine production by blood cel cultures. Arch Immunol Ther Exp (2000) 48:31–7. 10722229

[18] Barr JJ. A bacteriophages journey through the human body. Immunol Rev (2017) 279:106–22. 10.1111/imr.12565 28856733

[19] Borysowski J Górski A. Is phage therapy acceptable in the immunocompromised host? Int J Infect Dis (2008) 12:466–71. 10.1016/j.ijid.2008.01.006 18400541

[20] Przerwa A Zimecki M Świtała-Jeleń K Dabrowska K Krawczyk E Łuczak M Effects of bacteriophages on free radical production and phagocytic functions. Med Microbiol Immunol (2006) 195:143–50. 10.1007/s00430-006-0011-4 16447074

[21] D’Herelle F The Bacteriophage: Its Role in Immunity. Baltimore: Cornell University Library (1922).

[22] Jończyk-Matysiak E Łusiak-Szelachowska M Kłak M Bubak B Międzybrodzki R Weber-Dabrowska B The effect of bacteriophages preparations on intracellular killing of bacteria by phagocytes. J Immunol Res (2015) 2015:482863 10.1155/2015/482863 26783541 PMC4689956

[23] Ottonello L Dapino P Pastorino G Dallegri F Sacchetti C. Neutrophil dysfunction and increased susceptibility to infection. Eur J Clin Invest (1995) 25:687–92. 10.1111/j.1365-2362.1995.tb01987.x 7498244

[24] Sarantis H Grinstein S Subversion of phagocytosis for pathogen survivial. Cell Host Microbe (2012) 12:419–31. 10.1016/j.chom.2012.09.001 23084912

[25] Roach DR Leung CY Henry M Morello E Singh D Di Santo JP Synergy between the host immune system and bacteriophage is essential for successful phage therapy against an acute respiratory pathogen. Cell Host Microbe (2017) 22:38–47. 10.1016/j.chom.2017.06.018 28704651

[26] Xing L Genhua M Chunmei S Liangliang Z Lei H Qin J Role of M2 macrophages in sepsis-induced acute kidney injury. Shock (2017). 10.1097/SHK000000000000100628953574

[27] Lewkowicz N Mycko MP Przygodzka P Ćwiklińska H Cichalewska M Matysiak M Induction of human IL-10-producing neutrophils by LPS-stimulated Treg cells and IL-10. Mucosal Immunol (2016) 9:364–78. 10.1038/mi.2015.66 26220165

[28] Buchwald UK Geerdes-Fenge HF Vöckler J Ziege S Lode H. Interleukin-10: effects on phagocytosis and adhesion molecule expression of granulocytes and monocytes in a comparison with prednisolone. Eur J Med Res (1999) 4:85–94. 10085274

[29] Laichalk LL Danforth JM Standiford TJ. Interleukin-10 inhibits neutrophil phagocytic and bactericidal activity. FEMS Immunol Med Microbiol (1996) 15:181–7. 10.1111/j.1574-695X.1996.tb00084.x 8908479

[30] Lingnau M Höflich C Volk HD Sabat R Döcke WD. Interleukin-10 enhances the CD14-dependent phagocytosis of bacteria and apoptotic cells by human monocytes. Hum Immunol (2007) 68:730–8. 10.1016/j.humimm.2007.06.004 17869646

[31] Leech JM Lacey KA Mulcahy ME Medina E McLoughlin RM. IL-10 plays opposing roles during *Staphylococcus aureus* systemic and localized infections. J Immunol (2017) 198:2352–65. 10.4049/jimmunol.1601018 28167629 PMC5337812

[32] Deng LY Yang ZC Gong YL Huang GT Yin SP Jiang B Therapeutic effect of phages on extensively drug-resistant *Acinetobacter baumannii*-induced sepsis in mice. Zhonghua Shao Shang Za Zhi (2016) 32:523–8. 10.3760/cma.j.issn.1009-2587.2016.09.003 27647067

[33] Takemura-Uchiyama I Uchiyama J Osanai M Morimoto N Asagiri T Ujihara T Experimental phage therapy against lethal lung-derived septicemia caused by *Staphylococcus aureus* in mice. Microbes Infect (2014) 16:512–7. 10.1016/j.micinf.2014.02.011 24631574

[34] Hung CH Kuo CF Wang CH Wu CM Tsao N. Experimental phage therapy in treating *Klebsiella pneumoniae*-mediated liver abscesses and bacteremia in mice. Antimicrob Agents Chemother (2011) 55:1358–65. 10.1128/AAC.01123-10 21245450 PMC3067190

[35] Sunagar R Patil SA Chandrakanth RK. Bacteriophage therapy for *Staphylococcus aureus* bacteremia in streptozotocin-induced diabetic mice. Res Microbiol (2010) 161:854–60. 10.1016/j.resmic.2010.09.011 20868746

[36] Vinodkumar CS Kalsurmath S Neelagund YF. Utility of lytic bacteriophage in the treatment of multidrug-resistant *Pseudomonas aeruginosa* septicemia in mice. Indian J Pathol Microbiol (2008) 51:360–6. 10.4103/0377-4929.42511 18723958

[37] Watanabe R Matsumoto T Sano G Ishii Y Tateda K Sumiyama Y Efficacy of bacteriophage therapy against gut-derived sepsis caused by *Pseudomonas aeruginosa* in mice. Anitmicrob Agents Chemother (2007) 51:446–52. 10.1128/AAC.00635-06 PMC1797723 17116686

[38] Wang J Hu B Xu M Yan Q Liu S Zhu X Use of bacteriophage in the treatment of experimental animal bacteremia from imipenem-resistant *Pseudomonas aeruginosa*. Int J Mol Med (2006) 17:309–17. 10.3892/ijmm.17.2.309 16391831

[39] Wang J Hu B Xu M Yan Q Liu S Zhu X Therapeutic effectiveness of bacteriophages in the rescue of mice with extended spectrum beta-lactamase-producing *Escherichia coli* bacteremia. Int J Mol Med (2006) 17:347–55. 10.3892/ijmm.17.2.347 16391836

[40] Vinodkumar CS Neelagund YE Kalsurmath S. Bacteriophage in the treatment of experimental septicemic mice from a clinical isolate of multidrug resistant *Klebsiella pneumoniae*. J Commun Dis (2005) 37:18–29. 16637396

[41] Pouillot F Chomton M Blois H Courroux C Noelig J Bidet P Efficacy of bacteriophage therapy in experimental sepsis and meningitis caused by a clone O25b:H4-ST131 *Escherichia coli* strain producing CTX-M-15. Antimicrob Agents Chemother (2012) 56:3568–75. 10.1128/AAC.06330-11 22491690 PMC3393425

[42] Barrow P Lovell M Berchieri A Jr. Use of lytic bacteriophage for control of experimental *Escherichia coli* septicemia and meningitis in chickens and calves. Clin Diagn Lab Immunol (1998) 5:294–8. 9605979 10.1128/cdli.5.3.294-298.1998 PMC104512

[43] Diez-Martinez R De Paz HD Garcia-Fernandez E Bustamante N Euler CW Fischetti VA A novel chimeric phage lysin with high *in vitro* and *in vivo* bactericidal activity against *Streptococcus pneumoniae*. J Antimicrob Chemother (2015) 70:1763–73. 10.1093/jac/dkv038 25733585

[44] Schmelcher M Shen Y Nelson DC Eugster MR Eichenseher F Hanke DC Evolutionarily distinct bacteriophage endolysins featuring conserved peptidoglycan cleavage sites protect mice from MRSA infection. J Antimicrob Chemother (2015) 70:1453–65. 10.1093/jac/dku552 25630640 PMC4398471

[45] Gilmer DB Schmitz JE Euler CW Fischetti VA. Novel bacteriophage lysin with broad lytic activity protects against mixed infection by *Streptococcus pyogenes* and methicillin-resistant *Staphylococcus aureus*. Antimicrob Agents Chemother (2013) 57:2743–50. 10.1128/AAC.02526-12 23571534 PMC3716137

[46] Schuch R Lee HM Schneider BC Sauve KL Law C Khan BK Combination therapy with lysin CF-301 and antibiotic is superior to antibiotic alone for treating methicillin-resistant *Staphylococcus aureus*–induced murine bacteremia. J Infect Dis (2014) 209:1469–78. 10.1093/infdis/jit637 24286983 PMC3982849

[47] Vouillamoz J Entenza JM Giddey M Fischetti VA Moreillon P Resch G. Bactericidal synergism between daptomycin and the phage lysin CPl-1 in a mouse model of pneumococcal bacteraemia. Int J Antimicrob Agents (2013) 42:416–21. 10.1016/j.ijantimicag.2013.06.020 23992647

[48] Gu J Xu W Lei L Huang J Feng X Sun C LysGH15, a novel bacteriophage lysin, protects a murine bacteremia model efficiently against lethal methicillin-resistant *Staphylococcus aureus* infection. J Clin Microbiol (2011) 49:111–7. 10.1128/JCM.01144-10 21048011 PMC3020447

[49] Jado I Lopez R Garcia E Fenoll A Casal J Garcia P. Phage lytic enzymes as therapy for antibiotic-resistant *Streptococcus pneumoniae* infection in a murine sepsis model. J Antimicrob Chemother (2003) 52:967–73. 10.1093/jac/dkg485 14613958

[50] Weber-Dabrowska B Mulczyk M Górski A Bacteriophages as an efficient therapy for antibiotic-resistant septicemia in man. Transplant Proc (2003) 35:1385–6. 10.1016/S0041-1345(03)00525-6 12826166

[51] Schooley RT Biswas B Gill JJ Hernandez-Morales A Lancaster J Lessor L Development and use of personalized bacteriophage-based therapeutic cocktails to treat a patient with a disseminated resistant *Acinetobacter baumannii* infection. Anitmicrob Agents Chemother (2017) 61 10.1128/AAC.00954-17 PMC5610518 28807909

[52] Leitner L Sybesma W Chanishvili N Goderdzishvili M Chkhotua A Ujmajuridze A Bacteriophages for treating urinary tract infections in patients undergoing transurethral resection of the prostate: a randomized, placebo-controlled, double-blind clinical trial. BMC Urol (2017) 17:90. 10.1186/s12894-017-0283-6 28950849 PMC5615798

